# Relationships between X-ray Diffraction Peaks, Molecular Components, and Heat Properties of C-Type Starches from Different Sweet Potato Varieties

**DOI:** 10.3390/molecules27113385

**Published:** 2022-05-24

**Authors:** Yibo Li, Lingxiao Zhao, Lingshang Lin, Enpeng Li, Qinghe Cao, Cunxu Wei

**Affiliations:** 1 Key Laboratory of Crop Genetics and Physiology of Jiangsu Province/Joint International Research Laboratory of Agriculture & Agri-Product Safety of the Ministry of Education, Yangzhou University, Yangzhou 225009, China; dx120180133@yzu.edu.cn (Y.L.); 007520@yzu.edu.cn (L.L.); lep@yzu.edu.cn (E.L.); 2 Co-Innovation Center for Modern Production Technology of Grain Crops of Jiangsu Province/Jiangsu Key Laboratory of Crop Genomics and Molecular Breeding, Yangzhou University, Yangzhou 225009, China; 3 Xuzhou Institute of Agricultural Sciences in Jiangsu Xuhuai District, Xuzhou 221131, China; zhaolxiao2019@163.com

**Keywords:** sweet potato, C-type starch, molecular component, heat properties, principal component analysis

## Abstract

C-type starches with different proportions of A- and B-type crystallinities have different intensities and crystallinities of X-ray diffraction peaks. In this study, the intensities and crystallinities of X-ray diffraction peaks, molecular components and heat properties of C-type starches were investigated in seven sweet potato varieties, and their relationships were analyzed. The intensity and crystallinity of a diffraction peak at 5.6° were significantly positively correlated to the DP6-12 branch-chains of amylopectin and significantly negatively correlated to the true amylose content (TAC) determined by concanavalin A precipitation, gelatinization temperature, gelatinization enthalpy, water solubility at 95 °C, and pasting temperature. The intensity of diffraction peaks at 15° and 23° were significantly positively correlated to the gelatinization temperature and pasting temperature and significantly negatively correlated to the pasting peak viscosity. The significantly positive relationships were detected between the crystallinity of a diffraction peak at 15° and the DP13-24 branch-chains of amylopectin, gelatinization conclusion temperature and water solubility, between the crystallinity of diffraction peak at 17–18° and the TAC, gelatinization onset temperature, water solubility and pasting temperature, between the crystallinity of a diffraction peak at 23° and the gelatinization conclusion temperature and pasting peak time, and between the total crystallinity and the TAC, gelatinization conclusion temperature, water solubility and pasting temperature. The score plot of principle component analysis showed that the molecular components and heat property parameters could differentiate the C-type starches and agreed with their characteristics of X-ray diffraction peaks. This study provides some references for the utilizations of C-type starches.

## 1. Introduction

Starches are semi-crystalline granules and have A- and B-type crystallinities [1,2]. The A- and B-type crystallinities have different properties. For example, the B-type crystallinity has higher resistance to hydrolysis and exhibits lower gelatinization temperature than the A-type crystallinity [3]. Native starches from botanical resources are usually divided into A-, B- and C-type according to their containing crystallinity types. The A- and B-type starches have only A- and B-type crystallinity, respectively, and the C-type starch contains both A- and B-type crystallinities [1,2]. Normal cereal crops have A-type starches, some tuberous and high-amylose crops have B-type starches, and some legume and rhizome crops have C-type starches [1,2]. The A- and B-type starches have been widely studied and utilized [4,5]. However, the C-type starches are less studied than the A- and B-type starches.

The C-type starches are more complex than A- and B-type starches, and can be further classified into C_A_-, C_C_- and C_B_-types according to the ratio of A- and B-type crystallinities from high to low [1,2]. In fact, the distributions of A- and B-type crystallinities in C-type starches are also different due to their different botanical resources [2]. The A- and B-type crystallinities are located in the outer and inner regions of C-type starch granules from pea, respectively [6], but they are distributed in the inner and outer regions of C-type starch granules from high-amylose rice with inhibition of starch branching enzymes, respectively [7]. For the C-type starch with eccentric hilum from lotus rhizome, the periphery and distal region of hilum have A- and B-type crystallinities, respectively, and the center of granule has a mixture of A- and B-type crystallinities [8]. The C-type starch from root tuber of *Apios fortunei* has A- and B-type starch granules, meaning that the A- and B-type crystallinities are distributed in the different granules [9]. The different distribution patterns of A- and B-type crystallinities even further increase the complexity of C-type starches.

The root tuber of sweet potato (*Ipomoea batatas*) has high starch content (about 15–30% wet weight and 50–80% dry weight) [10,11], and is an important starch resource. The C-type starches from root tubers have been widely reported in sweet potato varieties. Significantly different X-ray diffraction (XRD) patterns and different scanning calorimetric (DSC) thermograms are detected in C-type starches from sweet potato varieties, and reflect different proportions of A- and B-type crystallinities [10,12,13,14,15,16,17,18]. The growing soil with high temperature increases the accumulation of A-type crystallinity in root tuber [13]. The C-type starches from sweet potato varieties grown in the same condition have significantly different XRD patterns and DSC thermograms, indicating that the genotypes play a major role in determining the accumulation of A- and B-type crystallinities [10,14,17]. Guo et al. [14] fitted the DSC curve of sweet potato starch with wide gelatinization temperature range (ΔT) into three peaks, and concluded that sweet potato root tuber has B-, C- and A-type starches, corresponding to the fitted peak with low, middle, and high gelatinization temperature, respectively. The starch properties, especially crystalline structure, affect the utilizations of starches [2,11]. However, it is no way to separate the A-, B- and C-type starches from sweet potato root tubers in practical production. Though it is easy to evaluate the proportion of A- and B-type crystallinities in starches using XRD patterns [1,19], it is unclear whether there are significant relationships between X-ray diffraction peaks and physicochemical properties of C-type starches.

In this study, C-type starches were isolated from seven sweet potato varieties, the XRD patterns, molecular components, and heat properties were investigated, and their relationships were analyzed. The objective of this study was to provide some information for the utilizations of C-type starches through investing their XRD patterns.

## 2. Results and Discussion

### 2.1. X-ray Diffraction Peaks of C-Type Starches

**Figure 1:**
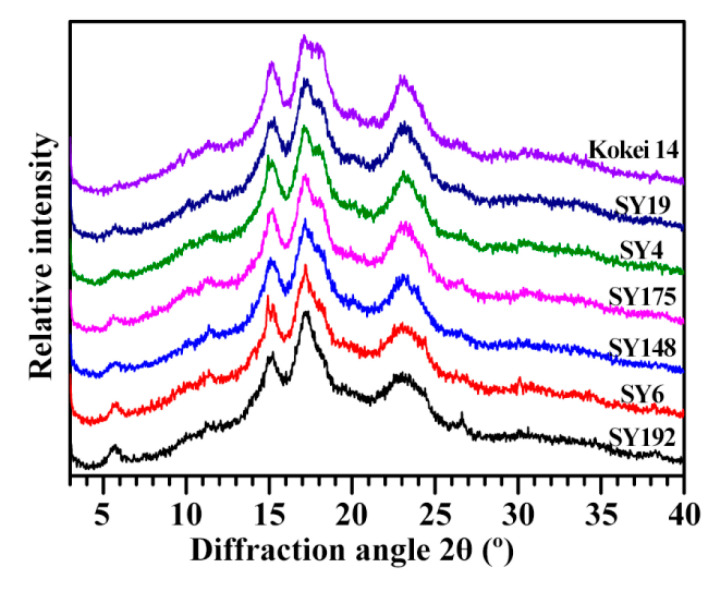
XRD patterns of C-type starches from different sweet potato varieties.

The starches from seven sweet potato varieties all exhibited C-type XRD patterns with diffraction peaks at 5.6°, 15°, 17° and 23° 2θ (Figure 1). The peaks at 5.6° and 23° are characteristic peaks of B- and A-type crystallinities, respectively [1,19]. In this study, the relative intensities of XRD patterns were normalized to the equal intensity (1000 counts) from the lowest point at 2θ about 4.3° to the highest point at 2θ 17–18°, resulting in that the intensities of diffraction peaks between different starches were at the same relative scale and therefore directly comparable. Though the seven starches all exhibited C-type XRD patterns, significant differences in the intensities of diffraction peaks were detected (Figure 1, Table 1). The intensity of diffraction peak decreased at 5.6° (from 146 to 38) and increased at 23° (from 592 to 727) from SY192 to SY6, SY148, SY175, SY4, SY19 and Kokei 14. In addition, the peak at 18°, a characteristic peak of A-type crystallinity [1,2], appeared as a shoulder peak in SY148 and its intensity increased from SY175 to SY4, SY19 and Kokei 14. The intensity variations of diffraction peaks indicated that the proportion of B-type crystallinity decreased and that of A-type crystallinity increased in C-type starch from SY192 to SY6, SY148, SY175, SY4, SY19 and Kokei 14. The C-type starches are usually divided into C_A_-, C_C_- and C_B_-type according to the ratio of A- to B-type crystallinity in C-type starch from high to low. The C_B_-type starch has diffraction peaks at 22° and 24°, and the C_A_-type starch has a diffraction peak at 18° [2]. The XRD patterns and peak intensities showed that the starch changed from C_C_-type to C_A_-type from SY192 to SY6, SY148, SY175, SY4, SY19 and Kokei 14 (Figure 1, Table 1).

Sweet potatoes have C_A_-, C_B_- and C_C_-type starches in different colored root tubers [10,13,14,15,16,17,18]. The types of C-type starches have no relationships with the color of root tuber, and are determined by the genotypes of sweet potato varieties [10,14,16,17]. Genkina et al. [13] concluded that the soil temperature affects the crystalline type of sweet potato starch with C_A_-type starch at soil temperature above 33 °C and C_B_-type starch at soil temperature below 15 °C. In fact, the temperature, the moisture and starch content in starch storage tissue and the branch-chain length of amylopectin all influence the crystal conformation during starch synthesis. The low temperature, high moisture, low starch content, and amylopectin long branch-chains tend to form B-type crystallinity, and the high temperature, low moisture, high starch content, and amylopectin short branch-chains tend to form A-type crystallinity [20]. The C-type starch from sweet potato grown at low soil temperature has higher ratio of B-type crystallinity and lower contents of starch and amylopectin long branch-chains than at high soil temperature [21], indicating that the temperature, moisture and starch content play more important roles in the formation of crystalline structure in sweet potato than the amylopectin structure. The relative crystallinities (RCs) of diffraction peaks and total RCs showed some differences among some sweet potato varieties (Table 1). The RC at 5.6°, 15°, 17–18° and 23° varied from 0.09% (Kokei 14) to 1.23% (SY192), from 3.50% (SY192) to 4.40% (SY4), from 9.27% (SY6) to 11.92% (Kokei 14) and from 8.15% (SY6) to 9.77% (SY175), respectively, and the RC at all crystalline diffraction peaks (RC_Total_) ranged from 21.96% (SY6) to 25.93% (SY19) among seven starches. The RC is affected by amylose content, amylopectin structure, crystallinity type, and granule size [1,12,22,23,24].

### 2.2. Molecular Components of C-Type Starches

The starch–iodine absorption spectrum was detected (data not shown). The data of optical density (OD) at 620 nm (OD620) and 550 nm (OD550) were obtained from the spectrum, and their ratio (OD620/550) reflects the relative proportion of long chains in starch [25]. The OD620/550 ranged from 1.146 (SY175) to 1.220 (SY4) among seven starches (Table 2), indicating that they had different amylose and amylopectin contents. The ratio of amylose and amylopectin is the most important structure parameter, determining the physicochemical properties and applications of starch [25,26]. The apparent amylose content (AAC) determined by iodine colorimetry usually overestimates the amylose content of starch due to the fact that the branch-chains of amylopectin can also bind the iodine. For concanavalin A (Con A) precipitation method, starch is completely dissolved into amylose and amylopectin. The Con A specifically binds and precipitates the amylopectin. The quality percentage of amylose to both amylose and amylopectin is usually defined as true amylose content (TAC) due to the fact that the amylose content is not influenced by the purity and moisture of starch and non-starch components (lipid and protein) [25]. Among seven starches, AAC and TAC ranged from 20.2% (SY6) to 29.2% (SY175) and from 14.7% (SY192) to 19.7% (Kokei 14), respectively, and the TAC was significantly lower than the AAC (Table 2).

**Figure 2:**
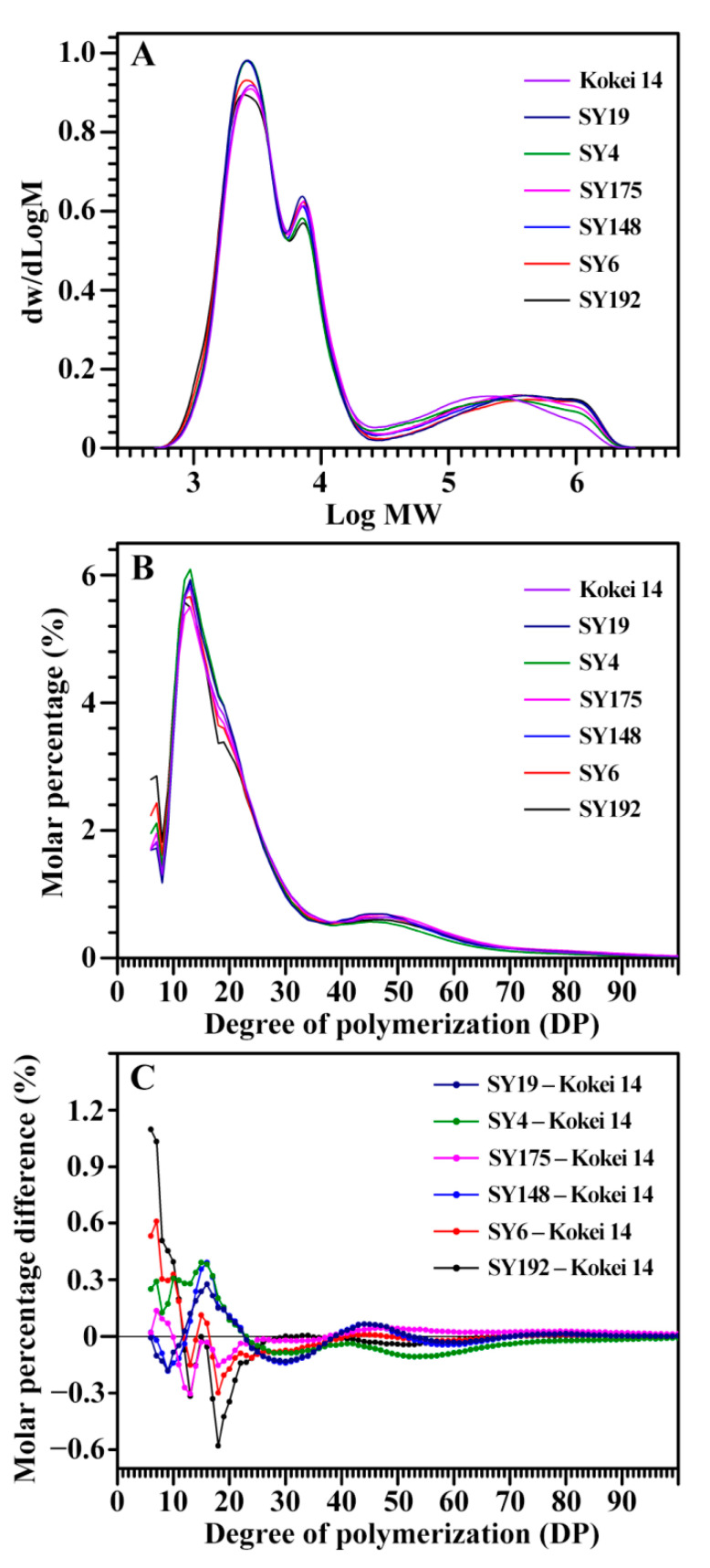
Gel permeation chromatography profiles of isoamylase-debranched starches (**A**) and fluorophore-assisted capillary electrophoresis profiles of isoamylase-debranched amylopectins (**B**,**C**) from different sweet potato varieties.

The molecular weight distribution of isoamylase-debranched starch is presented in Figure 2A. The peak of low, middle and high molecular weight presents the amylopectin short branch-chains (AP-S), amylopectin long branch-chains (AP-L), and amylose molecules (AM), respectively [24,25]. Their area percentages reflect the starch components, and the area ratio of low to middle molecular weight peak (AP-S/L) is positively correlated to the branching degree of amylopectin [24,25]. The seven sweet potato varieties had different starch components with AM from 18.7% (SY19) to 20.7% (SY192), AP-L from 21.5% (SY192) to 25.3% (SY19), AP-S from 55.1% (SY175) to 58.3% (SY4), and AP-S/L from 2.19 (SY175) to 2.69 (SY192) (Table 2). The chain length distribution of amylopectin was further analyzed (Figure 2B,C). Usually, amylopectin branch-chains are divided into A chains (DP6-12), B1 chains (DP13-24), B2 chains (DP25-36), and B3+ chains (DP > 36) [27]. The seven sweet potato starches exhibited different amylopectin chain length distributions with A chains from 20.2% (SY148) to 25.1% (SY192), B1 chains from 44.0% (SY192) to 49.0% (SY4), B2 chains from 12.7% (SY148) to 13.9% (SY192), B3+ chains from 15.6% (SY4) to 18.8% (SY175), and average branch-chain length (ACL) from 22.9 (SY4) to 24.6 DP (SY175) (Table 3).

In order to reveal the relationships between X-ray diffraction peaks and molecular components of C-type starches, the Pearson correlation coefficients between the intensities/crystallinities of X-ray diffraction peaks and the molecular components were evaluated (Table 4). The TAC was significantly positively correlated to the intensity of diffraction peak at 23° and the crystallinity of diffraction peak at 17–18° and significantly negatively correlated to the intensity and crystallinity of diffraction peak at 5.6°. The DP6-12 of amylopectin was significantly positively correlated to the intensity and crystallinity of diffraction peak at 5.6°. The DP13-24 of amylopectin was significantly positively correlated to the crystallinity of diffraction peak at 15° and significantly negatively correlated to the intensity of diffraction peak at 5.6° (Table 4). The DP6-12 of amylopectin is positively correlated to the ratio of B-type crystallinity in C-type starch from sweet potato grown at different soil temperatures [21], which is in line with the present study. For A- or B-type starches, the intensities and crystallinities of crystalline diffraction peaks are significantly negatively correlated to the amylose content and positively to the amylopectin short branch-chains [1,23,24]. However, Dhital et al. [12] separated maize A-type starch and potato B-type starch into different-sized fractions and found that the amylose content is significantly positively correlated to the RC of starch. The present results exhibited that C-type starches were more complex than A- and B-type starches in starch molecular components, especially for C-type starches with different proportions of A- and B-type crystallinities.

### 2.3. Heat Properties of C-Type Starches

**Figure 3:**
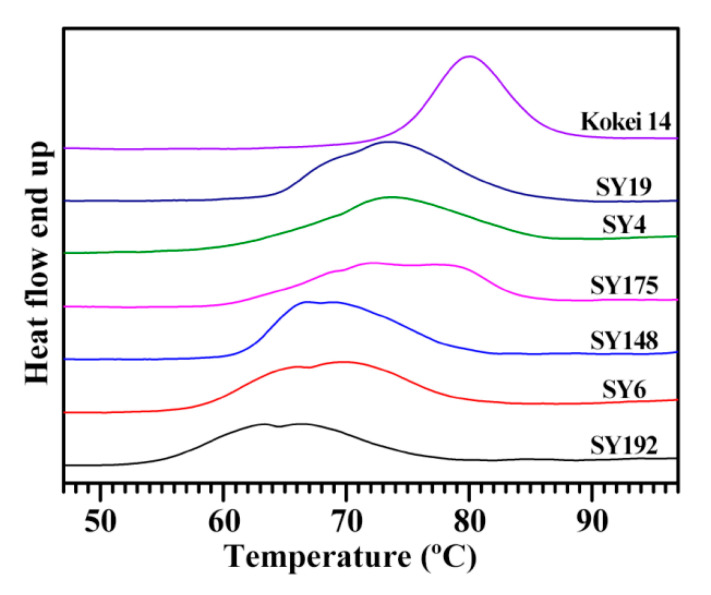
DSC thermograms of C-type starches from different sweet potato varieties.

The gelatinization properties of starch are analyzed with DSC. The DSC thermograms of seven starches exhibited significant differences in peak shapes, widths, and gelatinization temperatures (Figure 3, Table 5). Similar DSC thermograms are also reported in sweet potato starches from different varieties [15,28,29,30]. For example, Osundahunsi et al. [30] and Duan et al. [28] reported one- and two-peak thermograms of sweet potato starches, respectively, and Kim et al. [15] found a large variation in gelatinization temperature range (ΔT) from 13.0 to 36.7 °C among eight sweet potato starches. Genkina et al. [13] fitted the two-peak DSC curve of sweet potato starch into two gelatinization peaks with the low gelatinization temperature peak for B-type crystallinity and the high gelatinization temperature peak for A-type crystallinity. Guo et al. [14] fitted the DSC curve of sweet potato starch into three gelatinization peaks, and the peaks with low, middle and high gelatinization temperature correspond to the gelatinization of B-, C- and A-type starch, respectively, meaning that sweet potato root tuber contains A-, B- and C-type starches. In the present study, the gelatinization temperature and enthalpy (ΔH) exhibited significant differences. The Kokei 14 starch had the highest gelatinization temperature including onset (To), peak (Tp) and conclusion (Tc) temperature and the narrowest ΔT, and the SY192 starch had the lowest gelatinization temperature and ΔH among starches (Table 5). The sweet potato starches containing different proportions of A-, B- and C-type starches have different DSC thermograms [14]. The significantly different DSC thermograms of seven sweet potato starches might be due to their different proportions of A-, B- and C-type starches, and this agreed with their XRD patterns.

**Figure 4:**
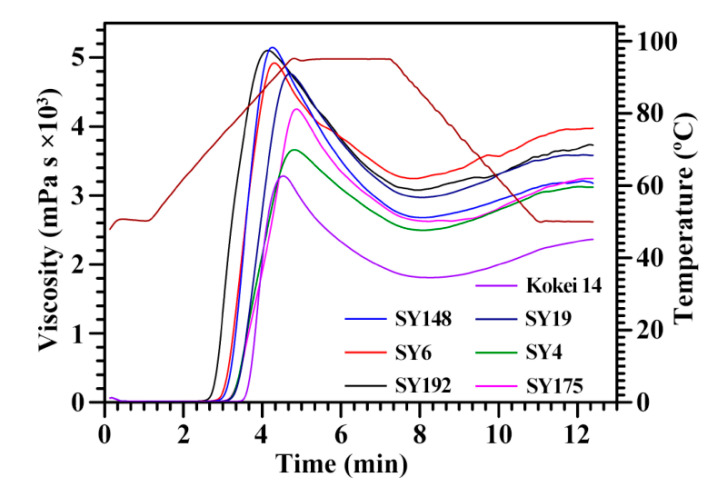
RVA profiles of C-type starches from different sweet potato varieties.

The swelling power (SP) and water solubility (WS) at 95 °C ranged from 24.0 (Kokei 14) to 27.6 g/g (SY148) and from 10.4% (SY192) to 12.4% (Kokei 14), respectively, among seven sweet potato starches (Table 5). Collado et al. [31] reported that the SP and WS vary from 24.5 to 32.7 g/g and from 12.1% to 24.1%, respectively, among 44 sweet potato starches. The pasting properties of starches were analyzed using a rapid visco analyzer (RVA) (Figure 4). The RVA profiles and pasting property parameters including pasting viscosity and temperature and peak time exhibited significant differences among seven sweet potato starches (Figure 4, Table 6). Kokei 14 starch had significantly lower peak (PV) (3265 mPa·s), hot (HV) (1789 mPa·s) and final viscosity (FV) (2341 mPa·s) and higher pasting temperature (P_Temp_) (81.4 °C) than the other starches, SY6 starch had the highest HV (3220 mPa·s) and FV (3993 mPa·s), and the SY148 starch had the highest PV (5152 mPa·s). The breakdown viscosity (BV) was the lowest for SY4 (1173 mPa·s) and the highest for SY148 (2503 mPa·s), and the setback viscosity (SV) was the lowest for SY148 (500 mPa·s) and the highest for SY6 (773 mPa·s). The peak time (P_Time_) was the lowest for SY192 (4.18 min). The PV and HV are the maximum and lowest viscosity of the heating starch paste, respectively, and they reflect the ability of binding water and granule swelling of starch [32]. The FV reflects the stability of cooled starch paste [33]. The BV (PV–HV) and SV (FV–HV) reflect the paste resistance to heat and shear and the paste retrogradation to cool, respectively, and the P_Time_ reflects the rate of starch swelling during heating [34]. The P_Temp_ is the critical temperature. Before the P_Temp_, starch granules in an excess of water continue to swell with increasing heat temperature, and mainly amylose but also some amylopectin leach out of the granules; after reaching the P_Temp_, starch granules begin to lose their granular shapes, and both the increased surface interactions between the swelling granules and the leached-out starch components will lead to a rise in viscosity [35]. The above RVA parameters are influenced by granule morphology and size, amylose content, crystalline structure, and starch purity [32,33,34]. Guo et al. [14] reported that the pasting properties of sweet potato starches have no relationship with the color of root tuber, and are determined by the genotypes of sweet potato varieties.

The Pearson correlations between intensities/crystallinities of X-ray diffraction peaks and heat property parameters of C-type starches were analyzed (Table 7). The gelatinization temperature and ΔH were significantly negatively correlated to the intensity and crystallinity of diffraction peak at 5.6°. The gelatinization temperature was significantly positively correlated to the intensity of diffraction peaks at 15° and 23°. In addition, the gelatinization conclusion temperature (Tc) was positively correlated to the crystallinity of diffraction peaks at 15° and 23°. The present results agreed with the previous report that B-type crystallinity has lower gelatinization temperature than A-type crystallinity [3,9]. The SP had no significant relationships with the intensity and crystallinity of crystalline diffraction peaks, but the WS was significantly positively correlated to the intensity of the diffraction peak at 23° and the crystallinity of diffraction peaks at 15° and 17–18°. For RVA parameters, the intensity of diffraction peak at 5.6° was significantly positively correlated to the HV and FV but significantly negatively correlated to the P_Temp_. The intensity of the diffraction peak at 15° was significantly negatively correlated to the PV, HV and FV, but significantly positively correlated to the P_Temp_. The intensity of diffraction peak at 23° was significantly negatively correlated to the PV and significantly positively correlated to the P_Time_ and P_Temp_. The total crystallinity was positively correlated to the P_Temp_, and the crystallinity of diffraction peak at 17–18° was negatively correlated to the FV and SV and positively correlated to the P_Temp_ (Table 7). For rice A-type starches, the RC is positively correlated to the To and negatively to the SP [23]. For high-amylose maize B-type starch, the RC is negatively correlated to the Tp, Tc and ΔT and positively to the ΔH, SP and WS [24]. Among different-sized fractions of starch, the total crystallinity is significantly positively correlated to the PV, HV and FV and significantly negatively correlated to the P_Temp_ and Tc for maize A-type starch, and significantly positively correlated to the ΔH and significantly negatively correlated to the Tp, Tc, HV and FV for potato B-type starch [12]. The present results show that the complex relationships between X-ray diffraction peaks and heat properties might be due to the different proportions of A- and B-type crystallinities in C-type starches.

### 2.4. Principal Component Analysis of Molecular Components and Heat Properties of C-Type Starches

**Figure 5:**
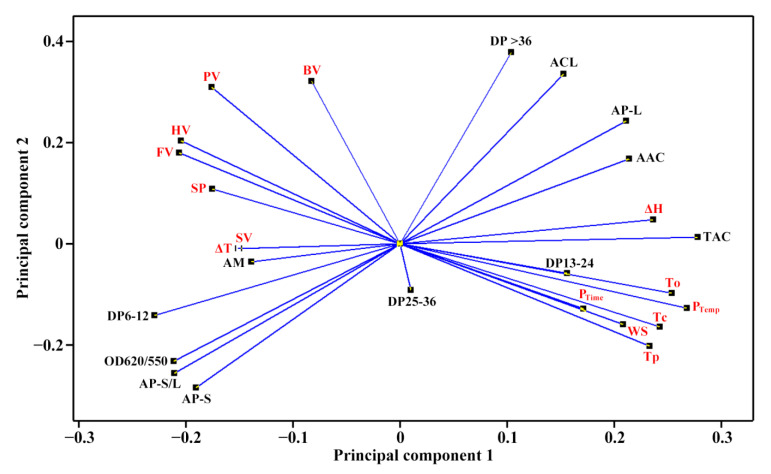
Loading plot of PCA based on the molecular components and heat properties of C-type starches. The abbreviations are listed in Table 2, Table 3, Table 5 and Table 6. The molecular components are in black, and the heat properties are in red.

**Figure 6:**
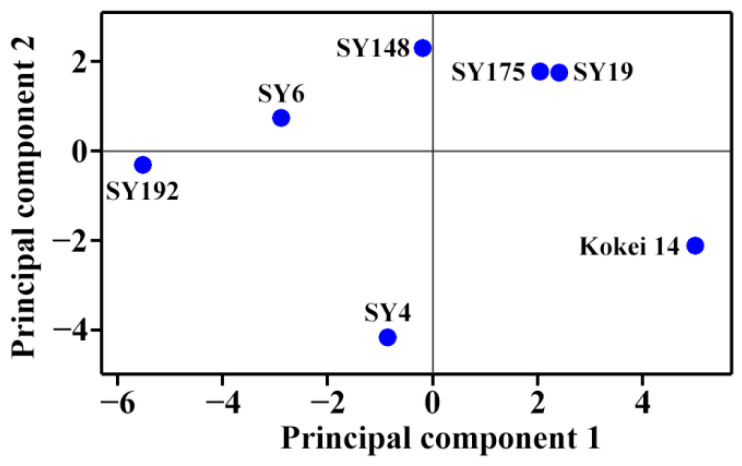
Score plot of PCA based on the molecular components and heat properties of C-type starches.

The molecular components (Table 2 and Table 3) and heat properties (Table 5 and Table 6) of seven sweet potato starches were subjected to principal component analysis (PCA) (Figure 5 and Figure 6). The first, second and third principal components could explain 47.8%, 21.8% and 13.6%, respectively, of the overall variation. The loading plot of PCA can exhibit the relationships among starch property parameters, and the score plot can show the similarities and differences among starches from different varieties [35]. In the present study, only the TAC was significantly positively correlated to the To, Tp, Tc, ΔH and P_Temp_ (Figure 5). Cai et al. [23] reported that the AP-S is significantly correlated negatively to the To, Tp, Tc and WS and positively to the SP, and the AM is significantly positively correlated to the To, Tp, Tc, ΔH and WS and significantly negatively correlated to the SP in rice A-type starches with different amylose contents. Lin et al. [24] reported that the To, Tp, Tc and ΔT are positively correlated to the AAC, AM and AP-L and negatively correlated to the AP-S, and the ΔH, SP and WS are correlated negatively to the AAC, AM and AP-L and positively to the AP-S in high-amylose maize B-type starch. In C-type starch from sweet potato, the DP6-12 of amylopectin is negatively correlated to the gelatinization temperature and pasting temperature, and positively correlated to the SV [21], which agrees with the present study. Among different-sized fractions of starch, the AM is significantly positively correlated to the PV, HV and FV and significantly negatively correlated to the P_Temp_ and Tc for maize A-type starch, and positively to the PV and ΔH and negatively to the Tp, Tc, HV and FV for potato B-type starch [12]. It is more complex for C-type starch to reveal the relationships between molecular components and heat properties, especially for sweet potato starch with a mixture of A-, B- and C-type starches. For P_Temp_, it was significantly positively correlated to gelatinization temperatures including To, Tp and Tc (Figure 5). Similar results have been reported in C-type starches from sweet potato [34] and A-type starches from endosperms of dicotyledon plants [36]. The score plot of PCA based on the molecular components and heat properties could differentiate the types of C-type starches (Figure 6), and agreed with their XRD patterns (Figure 1, Table 1). For example, Kokei 14 and SY192 were located at the far right and left of the score plot in principal component 1, respectively, indicating that they had significantly different properties and agreed with their X-ray diffraction peak characteristics.

## 3. Materials and Methods

### 3.1. Plant Materials

The 7 sweet potato varieties (SY4, SY6, SY19, SY148, SY175, SY192 and Kokei 14) were selected as plant materials due to the fact that their starches exhibited different C-type XRD patterns according to our previous study [37]. These germplasm resources are conserved in Xuzhou Sweetpotato Research Center, China. The SY4, SY6, SY19, SY148, SY175 and SY192 are origin accession IDs in China National Sweetpotato Genebank. The variety SY19 and SY192 originate from China, the variety SY148 and Kokei 14 originate from Japan, the variety SY4 and SY6 originate from the United States, and the variety SY175 originates from Peru. These sweet potato varieties were all planted simultaneously in the farm of Xuzhou Sweetpotato Research Center (32°27′ N, 117°29′ E), Jiangsu Province, China on 28 April, and harvested on 26 October 2020.

### 3.2. Isolation of Starch

Starches were isolated from fresh root tubers after harvest following the method of Guo et al. [10] with some modifications. Briefly, the pieces of root tubers in H_2_O were smashed and homogenized in a home blender. The starch-water slurry was filtered through 150, 75 and 50 μm sieves successively, and centrifuged (3000× *g*, 5 min) in a centrifuge (5430R, Eppendorf, Hamburg, Germany). The dirty surface layer above precipitated starch was removed off. The starch was washed successively with H_2_O (5 times) and anhydrous ethanol (3 times), and dried at 40 °C. The starch was filtered through 50 μm sieve, and stored at 4 °C.

### 3.3. Measurement of Starch–Iodine Absorption and Amylose Content

The starch–iodine absorption spectrum was analyzed following the procedures of Man et al. [22]. Briefly, starch was dissolved in dimethyl sulfoxide containing 10% 6.0 M urea (95 °C, 1 h). The dissolved amylose and amylopectin were colorized with iodine solution (0.2% I_2_ and 2% KI) in 50 mL volumetric flask containing 1 mL starch sample, 1 mL iodine solution, and 48 mL H_2_O. The sample was scanned using a spectrophotometer (BioMate 3S, Thermo Scientific, Chino, CA, USA). The absorbance data at 550 and 620 nm were obtained from the starch–iodine spectrum. The OD620 was used to measure the apparent amylose content (AAC). In order to avoid the effects of amylopectin and lipid on amylose content, the ratio of amylose to both amylose and amylopectin (usually defined as TAC) was determined with the concanavalin A precipitation method using an amylose/amylopectin assay kit (K-AMYL, Megazyme, Bray, Ireland).

### 3.4. Molecular Weight Distribution Analysis of Starch

The isoamylase-debranched starch was analyzed with a gel permeation chromatography (GPC) system (PL-GPC220, Agilent Technologies UK Limited, Shropshire, UK) following the procedures of Lin et al. [38] exactly. Briefly, the starch deproteinized by protease was dissolved in dimethyl sulphoxide (DMSO) solution, and then centrifuged (4000× *g*, 10 min). The supernatant was precipitated with anhydrous ethanol. The precipitated starch was dissolved and debranched with isoamylase (E-ISAMY, Megazyme, Bray, Ireland). The sample was freeze-dried. The dry starch powder was dissolved in DMSO and analyzed with GPC system with three columns of PL110-6100, 6300 and 6525 and a differential refractive index detector.

### 3.5. Chain Length Distribution Analysis of Amylopectin

The above isoamylase-debranched starch was labeled with the fluorophore APTS (8-amino-1,3,6-pyrenetrisulfonic acid) following the method of Lin et al. [27]. The sample was analyzed with a fluorophore-assisted capillary electrophoresis (FACE) system (PA800, Beckman-Coulter, Fullerton, CA, USA) following the procedures of Lin et al. [27].

### 3.6. Crystalline Structure Analysis of Starch

The dry starch powder was treated to absorb some moisture in a sealed container with a saturated NaCl solution for maintaining a 75% relative humidity at 25 °C for 10 d. The sample was detected using an X-ray diffractometer (D8, Bruker, Karlsruhe, Germany) at 40 kV, 40 mA, and 0.02° step size from 3° to 40° 2θ. The relative crystallinity (RC) was evaluated using the area percentage of crystalline peaks and total diffraction peaks between 4° to 30° 2θ following the method of Wei et al. [7].

### 3.7. Thermal Property Analysis of Starch

Five mg starch and 15 μL water were mixed and sealed in an aluminum pan. The sample was equilibrated at 4 °C overnight and 25 °C for 2 h before analysis, and detected using a differential scanning calorimeter (DSC200-F3, NETZSCH, Selb, Germany). The heating rate was 10 °C/min from 25 to 130 °C.

### 3.8. Measurement of Swelling Power and Water Solubility of Starch

The 2% (*v*/*v*) starch-water slurry was incubated in a ThermoMixer (1000 rpm, 95 °C, 30 min). The sample was centrifuged (5000× *g*, 10 min) after cooling at 25 °C for 10 min. The carbohydrates in the supernatant were quantified with an anthrone-H_2_SO_4_ method to measure the water solubility (WS), and the precipitation was weighed to measure the swelling power (SP) following the method of Guo et al. [10].

### 3.9. Pasting Property Analysis of Starch

Starch (2.5 g) and water (25 mL) slurry was analyzed using a rapid visco analyzer (3D, Newport Scientific, Warriewood, Australia). The programmed heating process included at 50 °C for 1 min, from 50 to 95 °C for 3.75 min (heating rate of 12 °C/min), at 95 °C for 2.5 min, from 95 to 50 °C for 3.75 min (cooling rate of 12 °C/min), and at 50 °C for 1.4 min. The pasting parameters including PV, HV, BV, FV, SV, P_Time_ and P_Temp_ were automatically calculated by the instrument software.

### 3.10. Principal Component Analysis (PCA)

The starch property parameters with significance of normal distribution over 0.05 were used for PCA with Minitab V.16.0 software (IBM Company, Chicago, IL, USA).

### 3.11. Statistical Analysis

The statistical differences, normal distribution, and Pearson correlation of data were evaluated with SPSS 16.0. Only the data with significance of normal distribution over 0.05 were used for Pearson correlation analysis.

## 4. Conclusions

The intensity of diffraction peak at 5.6° was significantly positively correlated to the DP6-12, HV and FV and significantly negatively correlated to the TAC, DP13-24, To, Tp, Tc, ΔH, WS and P_Temp_. The intensity of diffraction peak at 15° was significantly positively correlated to the To, Tp, Tc and P_Temp_ and significantly negatively correlated to the PV, HV and FV. The intensity of the diffraction peak at 23° was significantly positively correlated to the TAC, To, Tp, Tc, ΔH, WS, P_Time_ and P_Temp_ and significantly negatively correlated to the PV. The crystallinity of diffraction peak at 5.6° was significantly positively correlated to the DP6-12 and significantly negatively correlated to the TAC, To, Tp, Tc, ΔH, WS and P_Temp_. The crystallinity of diffraction peak at 15° was significantly positively correlated to the DP13-24, Tc and WS. The crystallinity of diffraction peak at 17–18° was significantly positively correlated to the TAC, To, WS and P_Temp_ and significantly negatively correlated to the FV and SV. The crystallinity of the diffraction peak at 23° was significantly positively correlated to the Tc and P_Time_. The total crystallinity was significantly positively correlated to the TAC, Tc, WS and P_Temp_. The score plot of PCA based on molecular components and heat properties could differentiate the type of C-type starches.

## Figures and Tables

**Table 1:** Relative intensities and crystallinities of diffraction peaks in XRD patterns of C-type starches.

Accession ID	RI of Diffraction Peak (Counts)			RC of Diffraction Peak (%)				
	RI_5.6°_	RI_15°_	RI_23°_	RC_5.6°_	RC_15°_	RC_17–18°_	RC_23°_	RC_Total_
SY192	146 ± 9 a	716 ± 6 d	592 ± 2 f	1.23 ± 0.18 a	3.50 ± 0.10 d	9.70 ± 0.14 de	8.35 ± 0.28 b	22.84 ± 0.31 cd
SY6	115 ± 7 b	761 ± 1 bc	617 ± 4 e	0.74 ± 0.08 b	3.63 ± 0.12 cd	9.27 ± 0.01 e	8.15 ± 0.22 b	21.96 ± 0.46 d
SY148	80 ± 1 c	751 ± 2 c	639 ± 2 d	0.56 ± 0.08 bc	3.94 ± 0.06 bc	11.17 ± 0.01 bc	8.35 ± 0.40 b	24.23 ± 0.65 bc
SY175	79 ± 2 c	778 ± 3 bc	676 ± 5 c	0.46 ± 0.04 bc	3.71 ± 0.21 cd	10.21 ± 0.25 d	9.77 ± 0.08 a	24.21 ± 0.41 bc
SY4	69 ± 2 c	788 ± 12 b	706 ± 8 b	0.34 ± 0.05 cd	4.40 ± 0.01 a	10.88 ± 0.08 c	9.02 ± 0.28 ab	24.84 ± 0.28 ab
SY19	67 ± 4 c	751 ± 15 c	708 ± 3 b	0.33 ± 0.10 cd	4.16 ± 0.10 ab	11.50 ± 0.33 ab	9.69 ± 0.20 a	25.93 ± 0.45 a
Kokei 14	38 ± 3 d	826 ± 6 a	727 ± 5 a	0.09 ± 0.01 d	4.20 ± 0.12 ab	11.92 ± 0.13 a	9.10 ± 0.41 ab	25.49 ± 0.34 ab
Sig.	0.558	0.906	0.547	0.516	0.713	0.830	0.275	0.723

RI: relative intensity; RC: relative crystallinity. The RIs of XRD patterns are normalized to the equal intensity (1000 counts) from the lowest point at 2θ about 4.3° to the highest point at 2θ about 17–18°. Sig.: the significance of normal distribution of the 7 samples by the Shapiro–Wilk test. Data are means ± standard deviations (*n* = 2). The values with different letters in the same column are significantly different (*p* < 0.05).

**Table 2:** Molecular components of C-type starches from different sweet potato varieties.

Accession ID	OD620/550	AAC (%)	TAC (%)	Components of Isoamylase-Debranched Starch			
				AM (%)	AP-L (%)	AP-S (%)	AP-S/L
SY192	1.208 ± 0.011 a	21.8 ± 0.2 c	14.7 ± 0.3 d	20.7 ± 0.1 a	21.5 ± 0.2 c	57.8 ± 0.3 ab	2.69 ± 0.04 a
SY6	1.203 ± 0.018 ab	20.2 ± 0.3 c	15.2 ± 0.7 d	19.3 ± 1.0 ab	23.8 ± 0.7 ab	56.9 ± 0.3 abc	2.39 ± 0.06 b
SY148	1.186 ± 0.001 abc	28.5 ± 0.2 a	17.2 ± 0.5 bc	19.9 ± 0.6 ab	23.9 ± 0.1 ab	56.3 ± 0.5 bcd	2.36 ± 0.01 b
SY175	1.146 ± 0.015 d	29.2 ± 1.3 a	17.7 ± 0.7 bc	19.8 ± 0.2 ab	25.1 ± 0.1 a	55.1 ± 0.3 d	2.19 ± 0.02 b
SY4	1.220 ± 0.018 a	21.5 ± 1.0 c	16.2 ± 0.1 cd	19.5 ± 0.3 ab	22.2 ± 0.1 bc	58.3 ± 0.3 a	2.62 ± 0.01 a
SY19	1.160 ± 0.012 cd	25.7 ± 0.8 b	18.2 ± 0.4 ab	18.7 ± 0.4 b	25.3 ± 0.9 a	56.0 ± 0.5 cd	2.21 ± 0.10 b
Kokei 14	1.169 ± 0.019 bcd	28.3 ± 0.2 a	19.7 ± 0.4 a	19.7 ± 0.3 ab	24.2 ± 1.1 ab	56.2 ± 0.9 bcd	2.33 ± 0.14 b
Sig.	0.787	0.156	0.925	0.871	0.440	0.810	0.331

OD620/550: absorption ratio of OD620 to OD550; AAC: apparent amylose content evaluated by the OD620 of starch–iodine absorption; TAC: true amylose content determined by the concanavalin A precipitation method; AM: amylose; AP-L: long branch-chains of amylopectin; AP-S: short branch-chains of amylopectin; AP-S/L: content ratio of short to long branch-chains of amylopectin. Sig.: the significance of normal distribution of the 7 samples by Shapiro–Wilk test. Data are means ± standard deviations (*n* = 3 for OD620/550 and AAC and = 2 for the others). The values with different letters in the same column are significantly different (*p* < 0.05).

**Table 3:** Chain length distributions of isoamylase-debranched amylopectins from C-type starches of different sweet potato varieties.

Accession ID	DP6-12 (%)	DP13-24 (%)	DP25-36 (%)	DP > 36 (%)	ACL (DP)
SY192	25.1 ± 0.5 a	44.0 ± 0.6 e	13.9 ± 0.1 a	17.0 ± 0.1 ab	23.5 ± 0.1 ab
SY6	23.5 ± 0.2 b	45.7 ± 0.4 d	13.0 ± 0.2 b	17.7 ± 0.4 ab	23.7 ± 0.1 ab
SY148	20.2 ± 0.5 d	48.9 ± 0.3 a	12.7 ± 0.1 b	18.1 ± 0.7 ab	24.1 ± 0.4 ab
SY175	21.1 ± 0.1 cd	46.2 ± 0.3 cd	13.8 ± 0.1 a	18.8 ± 0.4 a	24.6 ± 0.2 a
SY4	22.4 ± 0.7 bc	49.0 ± 0.6 a	13.0 ± 0.1 b	15.6 ± 1.4 b	22.9 ± 0.7 b
SY19	20.3 ± 0.4 d	48.4 ± 0.4 ab	12.8 ± 0.1 b	18.4 ± 0.7 a	24.3 ± 0.3 a
Kokei 14	21.4 ± 0.3 cd	47.4 ± 0.1 bc	13.8 ± 0.3 a	17.5 ± 0.2 ab	24.0 ± 0.1 ab
Sig.	0.438	0.467	0.066	0.583	0.924

ACL: average chain length of amylopectin. Sig.: the significance of normal distribution of the 7 samples by Shapiro–Wilk test. Data are means ± standard deviations (*n* = 2). The values with different letters in the same column are significantly different (*p* < 0.05).

**Table 4:** Pearson correlation coefficients between intensities/crystallinities of X-ray diffraction peaks and molecular components of C-type starches.

	RI_5.6°_	RI_15°_	RI_23°_	RC_5.6°_	RC_15°_	RC_17–18°_	RC_23°_	RC_Total_
OD620/550	0.486	−0.270	−0.431	0.466	−0.006	−0.393	−0.735	−0.490
AAC	−0.613	0.370	0.436	−0.521	0.175	0.614	0.524	0.574
TAC	−0.903 **	0.703	0.822 *	−0.856 *	0.571	0.855 *	0.651	0.824 *
AM	0.530	−0.306	−0.578	0.635	−0.533	−0.346	−0.448	−0.447
AP-L	−0.566	0.329	0.471	−0.601	0.144	0.376	0.615	0.440
AP-S	0.409	−0.232	−0.272	0.400	0.123	−0.263	−0.548	−0.301
AP-S/L	0.544	−0.324	−0.432	0.573	−0.091	−0.350	−0.596	−0.404
DP6-12	0.792 *	−0.397	−0.643	0.764 *	−0.547	−0.734	−0.570	−0.731
DP13-24	−0.764 *	0.424	0.676	−0.752	0.864 *	0.746	0.311	0.711
DP25-36	0.161	0.161	−0.068	0.209	−0.385	−0.179	0.160	−0.128
DP > 36	−0.094	−0.116	−0.044	−0.087	−0.384	0.042	0.335	0.067
ACL	−0.265	0.040	0.139	−0.241	−0.240	0.211	0.491	0.250

The abbreviations are listed in Table 1, Table 2 and Table 3. The * and ** indicate the significance at *p* < 0.05 and *p* < 0.01 level, respectively (*n* = 7).

**Table 5:** Thermal property parameters, swelling powers and water solubilities of C-type starches from different sweet potato varieties.

Accession ID	Thermal Property Parameters					SP (g/g)	WS (%)
	To (°C)	Tp (°C)	Tc (°C)	ΔT (°C)	ΔH (J/g)		
SY192	54.4 ± 0.2 f	65.8 ± 0.7 e	76.6 ± 1.2 d	22.2 ± 1.0 b	12.6 ± 0.3 c	26.2 ± 0.5 a	10.4 ± 0.8 b
SY6	57.9 ± 0.1 e	69.9 ± 0.1 d	79.9 ± 0.1 c	22.0 ± 0.1 b	14.9 ± 0.3 b	27.1 ± 0.3 a	10.7 ± 0.6 b
SY148	61.0 ± 0.4 c	66.8 ± 0.1 e	79.7 ± 0.1 c	18.7 ± 0.3 c	14.7 ± 0.3 b	27.6 ± 0.4 a	11.0 ± 0.2 ab
SY175	60.3 ± 0.1 d	72.5 ± 0.4 c	84.7 ± 0.6 b	24.4 ± 0.6 a	14.7 ± 0.2 b	24.5 ± 0.4 b	10.7 ± 0.1 b
SY4	60.3 ± 0.1 d	74.0 ± 0.3 b	85.8 ± 0.8 ab	25.5 ± 0.9 a	14.4 ± 0.3 b	26.4 ± 0.4 a	11.8 ± 0.4 ab
SY19	64.0 ± 0.1 b	73.6 ± 0.1 b	84.8 ± 0.4 b	20.8 ± 0.4 b	16.8 ± 0.5 a	26.4 ± 0.9 a	12.3 ± 0.6 a
Kokei 14	73.8 ± 0.1 a	80.0 ± 0.1 a	86.9 ± 0.1 a	13.1 ± 0.1 d	16.2 ± 0.1 a	24.0 ± 0.3 b	12.4 ± 0.8 a
Sig.	0.232	0.728	0.311	0.424	0.528	0.328	0.192

To: gelatinization onset temperature; Tp: gelatinization peak temperature; Tc: gelatinization conclusion temperature; ΔT: gelatinization temperature range (Tc–To); ΔH: gelatinization enthalpy; SP: swelling power; WS: water solubility. The SP and WS were determined at 95 °C. Sig.: the significance of normal distribution of the 7 samples by Shapiro–Wilk test. Data are means ± standard deviations (*n* = 2 for thermal property parameters and = 3 for SP and WS). The values with different letters in the same column are significantly different (*p* < 0.05).

**Table 6:** Pasting property parameters of C-type starches from different sweet potato varieties.

Accession ID	PV (mPa·s)	HV (mPa·s)	BV (mPa·s)	FV (mPa·s)	SV (mPa·s)	P_Time_ (min)	P_Temp_ (°C)
SY192	5140 ± 19 a	3062 ± 16 b	2078 ± 4 b	3722 ± 48 b	660 ± 65 b	4.18 ± 0.08 e	70.4 ± 0.5 d
SY6	4944 ± 51 b	3220 ± 59 a	1724 ± 9 d	3993 ± 42 a	773 ± 34 a	4.31 ± 0.08 d	73.6 ± 0.5 c
SY148	5152 ± 36 a	2648 ± 26 c	2503 ± 14 a	3148 ± 44 de	500 ± 35 d	4.27 ± 0.01 de	74.2 ± 0.1 c
SY175	4257 ± 52 c	2579 ± 35 c	1678 ± 29 d	3215 ± 27 d	636 ± 9 bc	4.82 ± 0.04 a	76.6 ± 0.1 b
SY4	3651 ± 31 d	2478 ± 13 d	1173 ± 18 f	3100 ± 36 e	622 ± 23 bc	4.82 ± 0.04 a	76.5 ± 0.1 b
SY19	4877 ± 58 b	3011 ± 42 b	1867 ± 32 c	3622 ± 37 c	611 ± 31 bc	4.67 ± 0.01 b	77.1 ± 0.5 b
Kokei 14	3265 ± 50 e	1789 ± 16 e	1476 ± 36 e	2341 ± 20 f	552 ± 6 cd	4.56 ± 0.04 c	81.4 ± 0.1 a
Sig.	0.139	0.439	0.980	0.658	0.816	0.293	0.857

PV: peak viscosity; HV: hot viscosity; BV: breakdown viscosity (PV–HV); FV: final viscosity; SV: setback viscosity (FV–HV); P_Time_: peak time; P_Temp_: pasting temperature. Sig.: the significance of normal distribution of the 7 samples by Shapiro–Wilk test. Data are means ± standard deviations (*n* = 3). The values with different letters in the same column are significantly different (*p* < 0.05).

**Table 7:** Pearson correlation coefficients between intensities/crystallinities of X-ray diffraction peaks and heat properties of C-type starches.

	RI_5.6°_	RI_15°_	RI_23°_	RC_5.6°_	RC_15°_	RC_17–18°_	RC_23°_	RC_Total_
To	−0.879 **	0.819 *	0.804 *	−0.844 *	0.631	0.827 *	0.419	0.718
Tp	−0.834 *	0.900 **	0.902 **	−0.860 *	0.682	0.635	0.597	0.670
Tc	−0.904 **	0.845 *	0.974 **	−0.934 **	0.784 *	0.672	0.766 *	0.782 *
ΔT	0.458	−0.424	−0.280	0.378	−0.201	−0.599	0.097	−0.333
ΔH	−0.793 *	0.560	0.760 *	−0.832 *	0.596	0.679	0.543	0.682
SP	0.457	−0.641	−0.534	0.433	−0.122	−0.305	−0.609	−0.390
WS	−0.825 *	0.608	0.883 **	−0.816 *	0.879 **	0.870 *	0.490	0.862 *
PV	0.716	−0.918 **	−0.779 *	0.721	−0.651	−0.500	−0.433	−0.517
HV	0.779 *	−0.854 *	−0.698	0.708	−0.584	−0.705	−0.329	−0.598
BV	0.397	−0.672	−0.601	0.486	−0.501	−0.095	−0.400	−0.246
FV	0.789 *	−0.803 *	−0.691	0.703	−0.601	−0.759 *	−0.330	−0.643
SV	0.596	−0.263	−0.433	0.456	−0.506	−0.822 *	−0.226	−0.690
P_Time_	−0.650	0.579	0.799 *	−0.702	0.608	0.377	0.857 *	0.628
P_Temp_	−0.950 **	0.907 **	0.933 **	−0.953 **	0.721	0.770 *	0.625	0.766 *

The abbreviations are listed in Table 1, Table 5 and Table 6. The * and ** indicate the significance at *p* < 0.05 and *p* < 0.01 level, respectively (*n* = 7).

## Data Availability

The data are available upon request from the corresponding author.

## Sample Availability

Samples of some starches are available from the corresponding author upon reasonable request.
